# Electronic and Structural Effects in Porphyrin Metal–Organic Framework Sensitizers for Solid‐State Dye‐Sensitized Solar Cells

**DOI:** 10.1002/cphc.70508

**Published:** 2026-07-29

**Authors:** Tommy Taing, Jesus Corona, Sailaja Muduganti, Xiyuan Yao, Matthew J. Tang, Doroteo Manriquez, Crystal Xiong, Oscar O. Bernal, Lun An, Long Qi, Dianlu Jiang, Yangyang Liu

**Affiliations:** ^1^ Department of Chemistry and Biochemistry California State University Los Angeles California USA; ^2^ Department of Physics and Astronomy California State University Los Angeles California USA; ^3^ U.S. DOE Ames National Laboratory Iowa State University Ames Iowa USA

**Keywords:** dopant engineering, interfacial charge transfer, metal–organic frameworks, porphyrin sensitizers, recombination dynamics, solid‐state dye‐sensitized solar cells

## Abstract

Porphyrin metal–organic frameworks (PMOFs) offer structurally ordered and compositionally tunable platforms for sensitized photovoltaics. Titanium‐ and indium‐based PMOFs, together with a tin‐doped indium analog, were investigated as photosensitizers in solid‐state dye‐sensitized solar cells (ssDSSCs). Electrochemical and optical measurements confirmed favorable energy‐level alignment with the TiO_2_ conduction band and the hole‐transporting material, supporting charge injection and transport. Distinct crystallite morphologies between Ti‐ and In‐based PMOFs govern interfacial geometry and recombination pathways within the mesoporous TiO_2_ scaffold. In contrast, the enhanced performance of In‐PMOF(Sn) relative to In‐PMOF is attributed primarily to dopant‐induced modifications in redox behavior and interfacial charge‐transfer kinetics rather than substantial shifts in frontier orbital energies. Although overall efficiencies remain modest, these findings establish clear structure–kinetics–performance relationships and demonstrate that node chemistry and dopant incorporation provide complementary strategies for tuning interfacial charge‐transfer dynamics in PMOF‐sensitized ssDSSCs.

## Graphical abstract

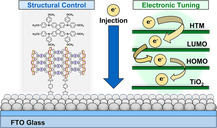

## 1 Introduction

The continued reliance on fossil fuels has accelerated greenhouse gas emissions, contributing to climate instability and environmental degradation [1, 2]. In response, renewable energy technologies have gained significant attention, with solar energy emerging as one of the most promising sustainable power sources due to its abundance and scalability [1, 3]. One of the most widely used methods for harnessing solar energy is photovoltaic technology, which converts sunlight directly to electricity through solar cells [1, 4, 5]. Researchers have investigated various types of solar cells, including silicon‐based, thin‐film, perovskite, organic, and dye‐sensitized solar cells (DSSCs) [1, 3, 4, 6, 7]. Among these technologies, DSSCs have attracted significant attention due to their low fabrication costs, mechanical flexibility, compatibility with roll‐to‐roll processing, and potential to achieve high power conversion efficiency (PCE) under both high‐intensity sunlight and diffuse lighting conditions [4, 5]. In particular, solid‐state DSSCs (ssDSSCs), which replace liquid electrolytes with hole‐transporting materials (HTMs), address issues related to electrolyte leakage and long‐term instability, thereby improving device robustness and integration potential [5, 7].

**FIGURE 1:**
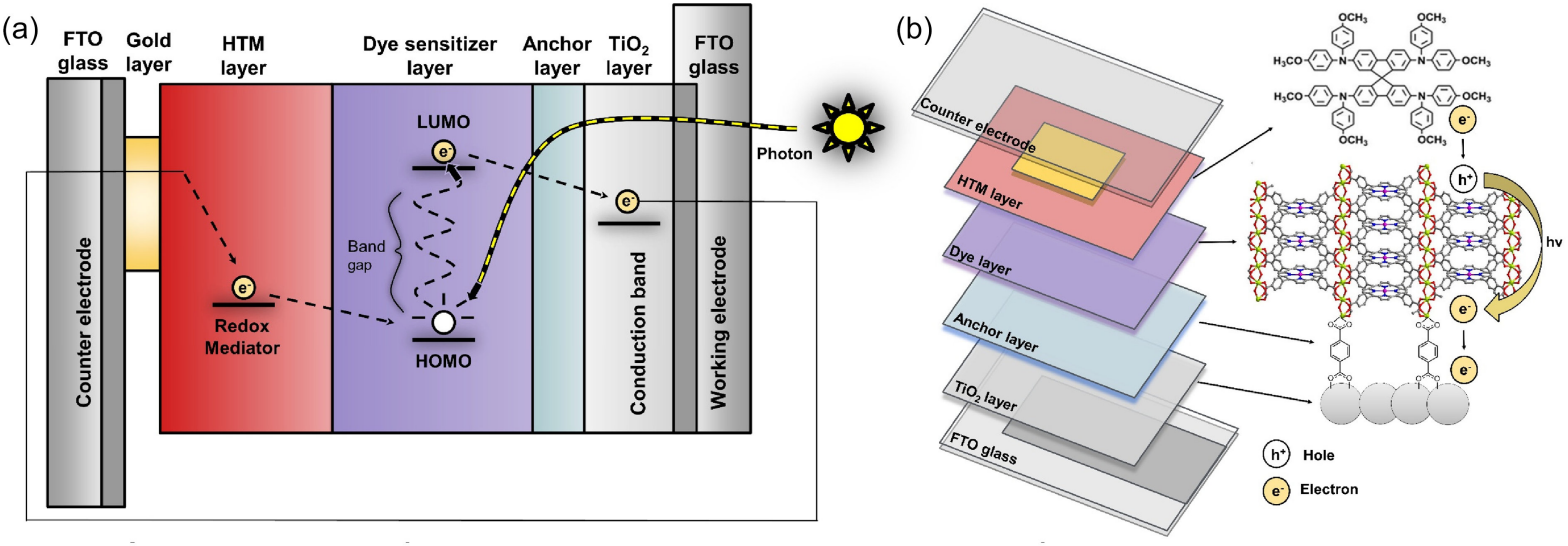
(a) Schematic illustration of the device architecture and operating mechanism of a typical solid‐state dye‐sensitized solar cell (ssDSSC), showing photoexcitation of the dye, electron injection into the TiO_2_ conduction band, dye regeneration by the hole‐transporting material (HTM), and charge collection at the electrodes. (b) Layered configuration of the ssDSSC incorporating porphyrin metal–organic framework (PMOF) sensitizers, including FTO substrate, TiO_2_ layers, anchoring layer, PMOF sensitizer layer, HTM, and gold counter electrode.

A typical ssDSSC consists of a working electrode, a counter electrode, a transparent conducting oxide (TCO)‐coated glass substrate, a photosensitive dye, and a redox mediator, all in a solid‐state configuration, as demonstrated in Figure 1a [4, 7, 8]. The working electrode, or photoanode, is composed of a wide‐bandgap semiconducting material, most commonly n‐type TiO_2_, deposited onto a TCO substrate in a uniform nanocrystalline structure. TiO_2_ is widely employed in DSSCs due to its chemical stability, nontoxicity, abundance, and low cost [1, 4, 7, 9]. However, its relatively large bandgap (3.2 eV) limits visible light absorption. To enhance light harvesting under both direct and diffuse illumination, TiO_2_ is sensitized with a molecular dye that absorbs photons in the visible region [9, 10]. The dye molecules are anchored to the TiO_2_ surface via coordinating functional groups that enable covalent attachment (anchor layer), ensuring efficient electronic coupling and charge transfer between the dye and the semiconductor (Figure 1b) [4, 7, 11].

Upon illumination, the dye absorbs photons and promotes electrons from its highest occupied molecular orbital (HOMO) to its lowest unoccupied molecular orbital (LUMO), as demonstrated in Figure 1a. The excited electrons are injected into the conduction band of the TiO_2_ photoanode and transported through the nanocrystalline network to the external circuit, while the oxidized dye is regenerated by electron donation from the HTM. The oxidized HTM is subsequently reduced at the counter electrode (gold or platinum), completing the electrochemical cycle [1, 4, 7]. Efficient device operation, therefore, requires favorable energetic alignment between the dye, TiO_2_, and HTM, as well as minimized interfacial recombination between injected electrons and oxidized species.

For decades, small‐molecule dyes, particularly ruthenium (Ru)‐based complexes, have dominated DSSC research due to their strong metal‐to‐ligand charge‐transfer (MLCT) transitions and tunable redox properties [12, 16]. However, their application in ssDSSCs presents intrinsic limitations. The discrete molecular nature of these dyes often leads to random surface orientation, incomplete surface coverage, and aggregation on TiO_2_, all of which can introduce interfacial trap states and promote charge recombination [1, 4, 15, 17]. In addition, Ru complexes suffer from high cost, limited sustainability due to metal scarcity, and relatively modest molar extinction coefficients compared to many organic chromophores [1, 4, 18]. In recent years, extensive efforts have been devoted to the molecular engineering of porphyrin sensitizers for DSSCs, resulting in substantial improvements in the performance of standalone porphyrin dyes and establishing porphyrins as highly effective photosensitizers [19, 24]. Despite these advances, some free porphyrin sensitizers remain susceptible to aggregation and structural disorder on the TiO_2_ surface, limiting overall device performance [25, 26]. Consequently, alternative sensitizer architectures that preserve the excellent light‐harvesting characteristics of porphyrins while providing greater structural organization and interfacial control are highly desirable.

Metal–organic frameworks (MOFs) have emerged as a promising strategy to address these limitations [27, 29]. Constructed from metal nodes and organic linkers, MOFs form crystalline, porous networks with high surface area, structural periodicity, and tunable electronic properties. Their ordered architecture can spatially organize photoactive chromophores, suppress aggregation, and promote more uniform electronic coupling and charge transport within the photoactive layer. Accordingly, MOFs have been incorporated into DSSCs as photoanode modifiers, counter electrode materials, and light‐harvesting sensitizers [27, 29].

Among the various MOF families, porphyrin‐based MOFs (PMOFs) are particularly attractive because they combine the exceptional visible‐light absorption of porphyrin chromophores with the structural order and compositional tunability of crystalline frameworks [21]. Recent studies have demonstrated that oriented PMOF thin films and PMOF sensitizers can generate photocurrent in both liquid‐ and ssDSSCs [30, 32]. Strategies such as epitaxial thin‐film growth, variation of metal‐node chemistry, and molecular engineering of porphyrin linkers have further improved photocurrent generation and photovoltaic performance [19, 33, 34].

Despite these advances, most reported systems have focused on Zn‐, Zr‐, Cu‐, or Ru‐based frameworks [18, 22, 35, 38]. Comparative investigations of alternative node chemistries and dopant‐modified porphyrin frameworks in ssDSSCs remain scarce, particularly with respect to correlating framework composition with interfacial energetics and recombination dynamics. In particular, titanium‐, and indium‐based PMOFs are comparatively underexplored as sensitizers in ssDSSCs, despite their potential compatibility with TiO_2_ photoanodes and their tunable electronic structures. Moreover, while dopant incorporation is widely used to tailor semiconductor properties, its role within PMOFs, particularly in modulating energetic alignment and recombination kinetics in solid‐state devices, has not been systematically examined.

Herein, we investigated titanium‐ and indium‐based PMOFs (Ti‐PMOF and In‐PMOF), together with a tin (Sn)‐doped indium PMOF analog (In‐PMOF(Sn) or CSLA‐10), as photosensitizers in ssDSSCs. These frameworks feature one‐dimensional metal–oxo chain motifs coordinated with the π‐conjugated porphyrin ligand tetrakis(4‐carboxyphenyl)porphyrin (TCPP), forming structurally ordered networks with electronically active porphyrin units (Figure 1b). Using combined electrochemical, optical, and electron lifetime analysis of PMOF‐sensitized ssDSSCs, we systematically examined how framework composition and dopant incorporation influence energy‐level alignment, interfacial recombination kinetics, and overall photovoltaic performance. This work establishes structure–kinetics–performance relationships in PMOF‐sensitized ssDSSCs and provides mechanistic insight into the rational design of framework‐based sensitizers.

## 2 Experimental Section

### 2.1 Materials and Instrumentation

Titanium (IV) *n*‐butoxide (99+%), *N*,*N′*‐dimethyl formamide (DMF, 99.8%), isopropanol (99.5%), anhydrous ethanol (99.5%), acetone (99.5%), acetonitrile (99.9%), tetra‐*n*‐butylammonium hexafluorophosphate (98%), ethanolamine (99%), nitric acid (69%–70%), chlorobenzene (99+%), 4‐‍aminobenzoic acid (99%), benzoic acid (99%), and indium(III) nitrate hydrate (99.9%) were purchased from Thermo Fisher Scientific. 2,2′,7,7′‐Tetrakis[*N*,]*N*‐di(4‐methoxyphenyl) amino]‐9,9’‐spirobifluorene (Spiro‐OMeTAD, 99%), tris(2‐(1H‐pyrazol‐1‐yl)‐4‐*tert*‐butylpyridine)cobalt(III) tri[bis(trifluoromethane)sulfonimide] (Co(III) TFSI salt, 98%), benzene‐1,4‐dicarboxylic acid (BDC, 98%), and potassium hydroxide (85%) were purchased from Sigma–Aldrich. TCPP (97%) was purchased from Frontier Scientific. Titanium(IV) isopropoxide (98%+) and lithium bis(trifluoromethanesulfonyl)imide (LiTFSI, 99%) were obtained from Acros Organics. All reagents were used as received without further purification. Instrumentation details are provided in the Supporting Information.

### 2.2 Syntheses of PMOFs

*Synthesis of Ti‐PMOF*. Ti‐PMOF was synthesized according to a previously reported procedure [39]. The titanium cluster (Ti_6_O_6_(OiPr)_6_(abz)_6_) (4 mg; see SI for synthesis), TCPP (15 mg), benzoic acid (200 mg), and DMF (3 mL) were combined in a 15 mL pressure vessel and sonicated for 20 min. The sealed vessel was then heated at 150 °C for 48 h. The resulting dark red crystalline solid was collected, washed with DMF (5 mL × 3), then with acetone (5 mL × 3), and dried under vacuum at 60 °C for 8 h. The MOF was subsequently activated under dynamic vacuum at 80 °C for 12 h before use.

*Synthesis of In‐PMOF and In‐PMOF*(*Sn*)*/CSLA‐10*. In‐PMOF and In‐PMOF(Sn)/CSLA‐10 were synthesized according to previously reported procedures [40, 41]. A 0.5 M aqueous solution of In(NO_3_)_3_·H_2_O (0.23 mL), DMF (2.17 mL), and either TCPP (45 mg, for In‐PMOF) or TCPP(Sn) (51.6 mg, for In‐PMOF(Sn)) were combined in a 15 mL pressure vessel and sonicated for 20 min. The sealed vessel was then heated at 120 °C for 48 h. The resulting purple crystalline solids were collected, washed with DMF (5 mL × 3), then with acetone (5 mL × 3), and dried under vacuum at 60 °C for 8 h. Both MOFs were subsequently activated under dynamic vacuum at 140 °C for 16 h before use.

### 2.3 Fabrication of ssDSSCs

**FIGURE 2:**
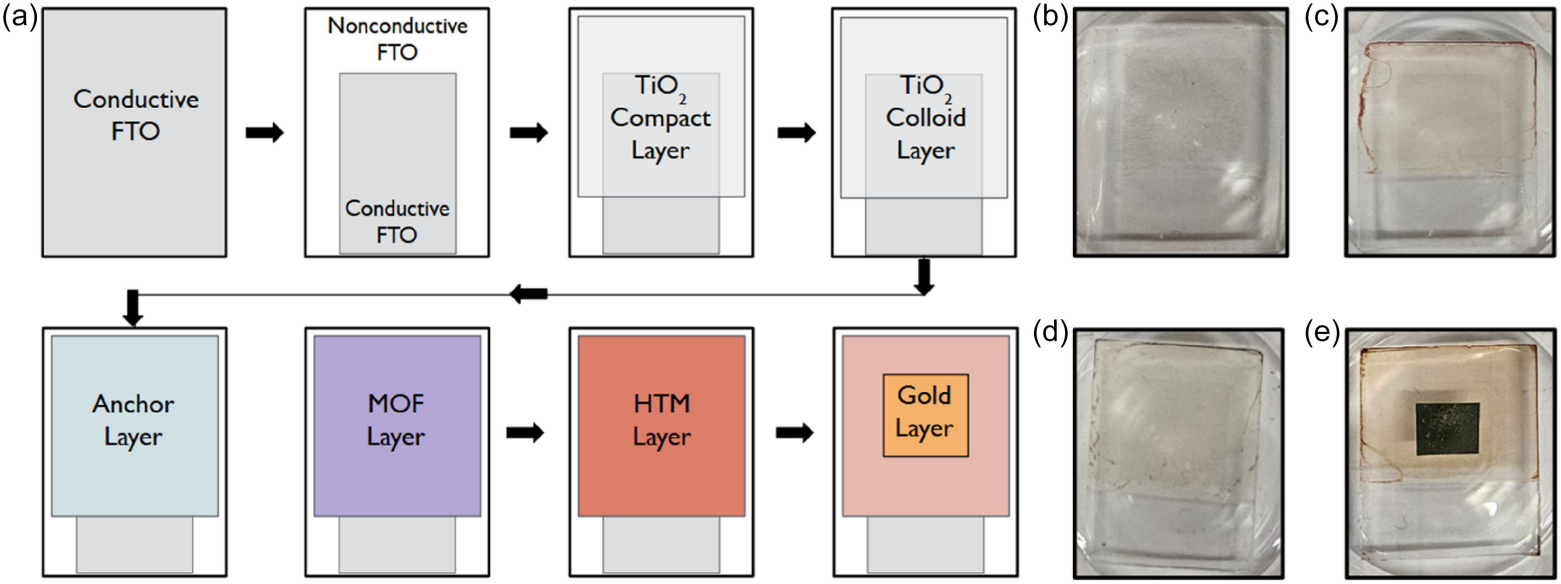
(a) Schematic illustration of the fabrication process for ssDSSCs incorporating porphyrin metal–organic framework (PMOF) sensitizers. (b–d) Photographs of devices after deposition of the PMOF layer and prior to HTM application; PMOF = Ti‐PMOF (b), In‐PMOF (c), and In‐PMOF(Sn) (d). (e) Photograph of a completed ssDSSC incorporating In‐PMOF after deposition of the HTM and sputtered gold counter electrode. Color legend: gray = conductive FTO surface; white = nonconductive glass; light gray = compact and mesoporous TiO_2_ layers; blue = anchoring layer; purple  = PMOF layer; dark orange = HTM; gold = counter electrode.

The fabrication procedure is schematically illustrated in Figure 2. Prior to device assembly, all precursor solutions used for the fabrication of individual solar cell layers were prepared according to the procedures described in the Supporting Information (Section S3).

*FTO Substrate Preparation.* Fluorine‐doped tin oxide (FTO) glass substrates (1.5 cm × 2 cm) were patterned to define the active electrode area and prevent short‐circuiting. Polyimide tape was applied to mask the working region, and the exposed FTO was chemically etched using 2 M HCl and zinc powder. The etched substrates were subsequently neutralized with potassium carbonate solution, and the tape was removed. The substrates were then sequentially cleaned using 1% detergent solution, isopropanol, acetone, and deionized water. After drying, the substrates were treated with oxygen plasma for 20 min prior to film deposition.

*Deposition of TiO*_2_ *Layers.* A compact TiO_2_ layer was deposited onto the cleaned FTO substrates by spin coating 0.3 mL of the precursor solution in a glovebox. The coated substrates were annealed at 500 °C for 12 h. After cooling to room temperature, a mesoporous TiO_2_ colloidal layer was spin‐coated onto substrates maintained at 70 °C, followed by a second annealing step at 500 °C for 12 h. The substrates were then allowed to cool to room temperature before further functionalization.

*Deposition of Anchor and PMOF Layers.* The TiO_2_‐coated substrates were immersed in a 0.01 M BDC anchoring solution at 60 °C for 24 h to promote surface coordination. The substrates were then rinsed with ethanol and air‐dried. PMOF suspensions (0.1 mM, calculated based on porphyrin centers) containing Ti‐PMOF, In‐PMOF, or In‐PMOF(Sn) were sonicated for 10 min immediately prior to deposition. The suspensions were then drop‐casted onto the anchored TiO_2_ surface in 30 μL increments with 10 min drying intervals between successive depositions. For optimization studies, total deposition volumes of 60, 90, 120, 150, and 180 μL were evaluated.

*Deposition of HTM and Counter Electrode.* The substrates bearing the TiO_2_, anchor, and MOF layers were transferred into a glovebox for deposition of the HTM. 50 μL of Spiro‐OMeTAD solution was spin‐coated onto each substrate. After drying, the coated substrates were removed from the glovebox and masked to define an active device area (0.5 cm × 0.5 cm). A gold electrode was then deposited by sputtering to complete the ssDSSC devices.

### 2.4 Photovoltaic Characterization

Photovoltaic measurements were performed using a CHI 610 electrochemical analyzer under simulated AM 1.5G illumination (137 mW cm^−2^). The ssDSSC device and a reference FTO glass substrate were mounted in a custom 3D‐printed holder, with the gold electrode of the device electrically connected to the conductive face of the reference FTO. Current density–voltage (*J*–*V*) characteristics were obtained by linear sweep voltammetry using a three‐electrode configuration.

PCE (*η*) was calculated according to Equation (1):

(1) $$\eta =\frac{V_{\mathrm{oc}}\times J_{\mathrm{sc}}\times \mathrm{FF}}{P_{\mathrm{in}}}$$

where *V*_oc_ is the open‐circuit voltage, *J*_sc_ is the short‐circuit current density, FF is the fill factor, and *P*_in_ is the incident light intensity [4].

Open‐circuit voltage decay (OCVD) measurements were performed under the same experimental configuration by interrupting the illumination. The electron lifetime (*τ*_*n*_) was estimated from the decay of the open‐circuit voltage according to Equation (2), as described by Zaban et al. [42], where *k*_*B*_ is the Boltzmann constant, *T* is the absolute temperature, *e* is the elementary charge of an electron, and *dV*_oc_ */dt* is the rate of voltage decay.

(2) $$\tau_{n}=-\frac{k_{B}T}{e}{\left(\frac{dV_{\mathrm{oc}}}{dt}\right)}^{-1}$$

## 3 Results and Discussion

### 3.1 PMOF Structures and Characterizations

**FIGURE 3:**
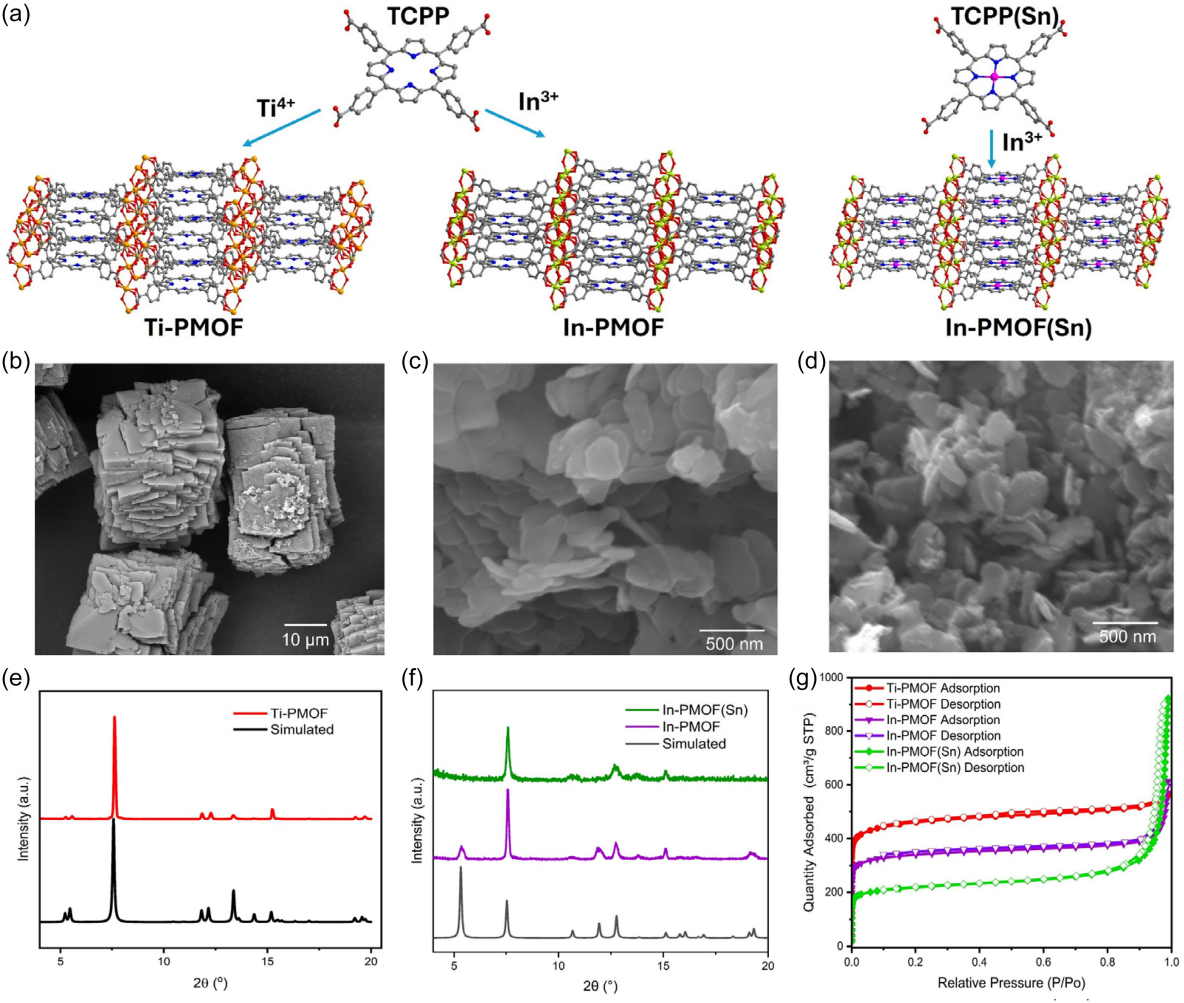
Structural and morphological characterization of the PMOFs. (a) Crystal structures of Ti‐PMOF, In‐PMOF, and In‐PMOF(Sn) constructed from TCPP or TCPP(Sn) ligands coordinated to Ti^4+^ or In^3+^ metal–oxo chains. (b–d) Scanning electron microscopy (SEM) images showing the morphology of Ti‐PMOF (b), In‐PMOF (c), and In‐PMOF(Sn) (d). (e) Powder X‐ray diffraction (PXRD) pattern of Ti‐PMOF compared with the simulated pattern. (f) PXRD patterns of In‐PMOF and In‐PMOF(Sn) compared with the simulated pattern. (g) N_2_ adsorption–desorption isotherms at 77 K for Ti‐PMOF (red), In‐PMOF (purple), and In‐PMOF(Sn) (green).

Three PMOFs, Ti‐PMOF, In‐PMOF, and the tin‐doped analog, In‐PMOF(Sn)/CSLA‐10, were synthesized via solvothermal methods adapted from prior reports [39, 41]. As shown in Figure 3a, the three PMOFs share similar architectures in which TCPP ligands coordinate to one‐dimensional metal‐oxo chains. The porphyrin units are arranged in alternating, staggered layers along the chains, separated by the metal‐oxo nodes, resulting in periodic spacing and directional organization of the chromophores. The Ti‐PMOF and In‐PMOF structures are constructed from Ti^4+^ or In^3+^ nodes linked by TCPP ligands, while in In‐PMOF(Sn), Sn is incorporated within the porphyrin core without altering the overall framework topology.

SEM analysis revealed distinct morphological differences (Figure 3b–d): Ti‐PMOF exhibited flake‐like cubic particles (∼15–20 µm), whereas In‐PMOF and In‐PMOF(Sn) formed smaller sheet‐like crystallites (∼300–500 nm). Such differences in particle size and morphology are expected to influence integration within the mesoporous TiO_2_ scaffold during drop‐casting. The smaller In‐based MOF crystallites may promote more uniform surface coverage and conformal contact, whereas the larger Ti‐PMOF flakes may generate less homogeneous interfacial domains. These variations in interfacial geometry are expected to influence charge injection and recombination behavior during device operation.

Powder X‐ray diffraction (PXRD) patterns of all three PMOFs were in good agreement with their simulated patterns, confirming phase purity and successful framework formation (Figure 3e,f). Minor variations in peak intensity, particularly for In‐PMOF and In‐PMOF(Sn), are attributed to preferred orientation effects, consistent with their sheet‐like morphology observed in SEM (Figure 3c,d). Nitrogen adsorption–desorption isotherms were measured at 77 K for three MOFs, confirming their permanent porosity following activation (Figure 3g). Topological analysis further confirms that Ti‐PMOF and In‐PMOF adopt the same underlying *sea* network topology (Figure S2), indicating that the overall framework connectivity is preserved despite differences in metal‐node composition.

### 3.2 Electrochemical and Optical Properties

**FIGURE 4:**
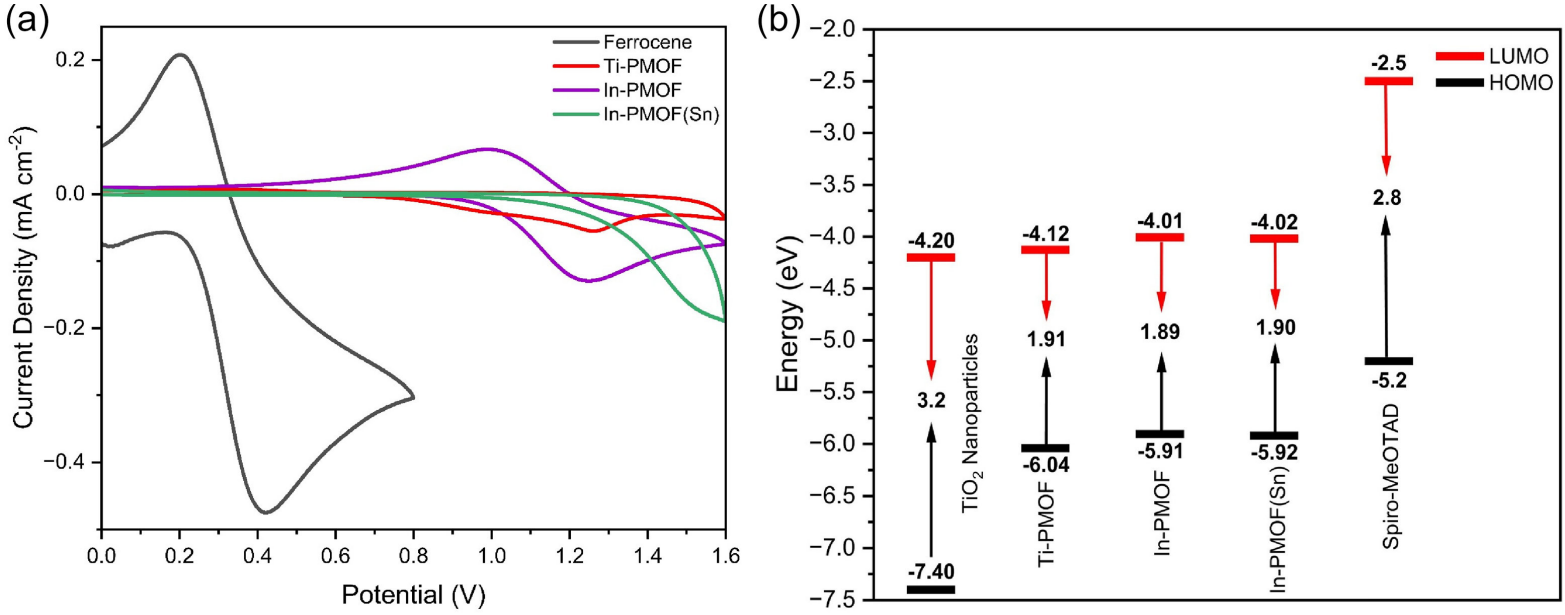
(a) Cyclic voltammograms of Ti‐PMOF, In‐PMOF, and In‐PMOF(Sn) films deposited on FTO, together with ferrocene (Fc/Fc^+^) as the reference, recorded in 1 M tetra‐*n*‐butylammonium hexafluorophosphate/acetonitrile electrolyte. (b) Estimated frontier orbital energy level diagram of Ti‐PMOF, In‐PMOF, and In‐PMOF(Sn), showing the HOMO and LUMO energy levels relative to the TiO_2_  band edges and the HOMO/LUMO energy levels of the hole‐transporting material (Spiro‐OMeTAD).

To evaluate the suitability of the PMOFs as photosensitizers in ssDSSCs, cyclic voltammetry (CV) and UV–vis spectroscopy were performed. CV measurements of PMOF films deposited on FTO substrates revealed distinct redox responses associated with the porphyrin ligand and framework environment (Figure 4a). In‐PMOF displayed a quasireversible redox couple with oxidation and reduction peaks centered at 1.22 and 1.05 V (vs. a pseudo‐reference electrode), consistent with porphyrin‐based redox processes [43, 44]. Ti‐PMOF exhibited a pronounced oxidation peak at 1.27 V but lacked a corresponding reduction peak, likely due to slow anion intercalation within the framework. In contrast, In‐PMOF(Sn) exhibited broader and less defined redox features with an oxidation onset near 1.15 V, suggesting increased kinetic limitations or greater heterogeneity in the redox process following Sn incorporation.

Using CV data referenced to ferrocene (Figure 4a) and optical band gaps obtained from UV–vis spectra (Figure S1), HOMO and LUMO energy levels were estimated for the three PMOFs (details are described in the Supporting Information). The band gaps of Ti‐PMOF, In‐PMOF, and In‐PMOF(Sn) were calculated to be 1.91, 1.89, and 1.90 eV, respectively (Figure 4b). These values are significantly smaller than those of TiO_2_ (3.2 eV), enabling efficient absorption of visible light by the PMOF sensitizers and facilitating photoexcitation under solar illumination.

Importantly, the estimated LUMO levels of all PMOFs lie above the TiO_2_ conduction band edge (∼–4.2 eV), satisfying the energetic requirement for electron injection from the photoexcited porphyrin sensitizer into the TiO_2_ conduction band. Furthermore, the estimated HOMO levels of all PMOFs are positioned below that of the HTM, providing a sufficient driving force for dye regeneration via hole transfer from the oxidized sensitizer to the HTM. Upon Sn incorporation, only minimal changes in the estimated frontier orbital energies were observed, indicating that the overall electronic structure of the porphyrin framework is largely preserved.

### 3.3 Device Fabrication and Photovoltaic Performance

**FIGURE 5:**
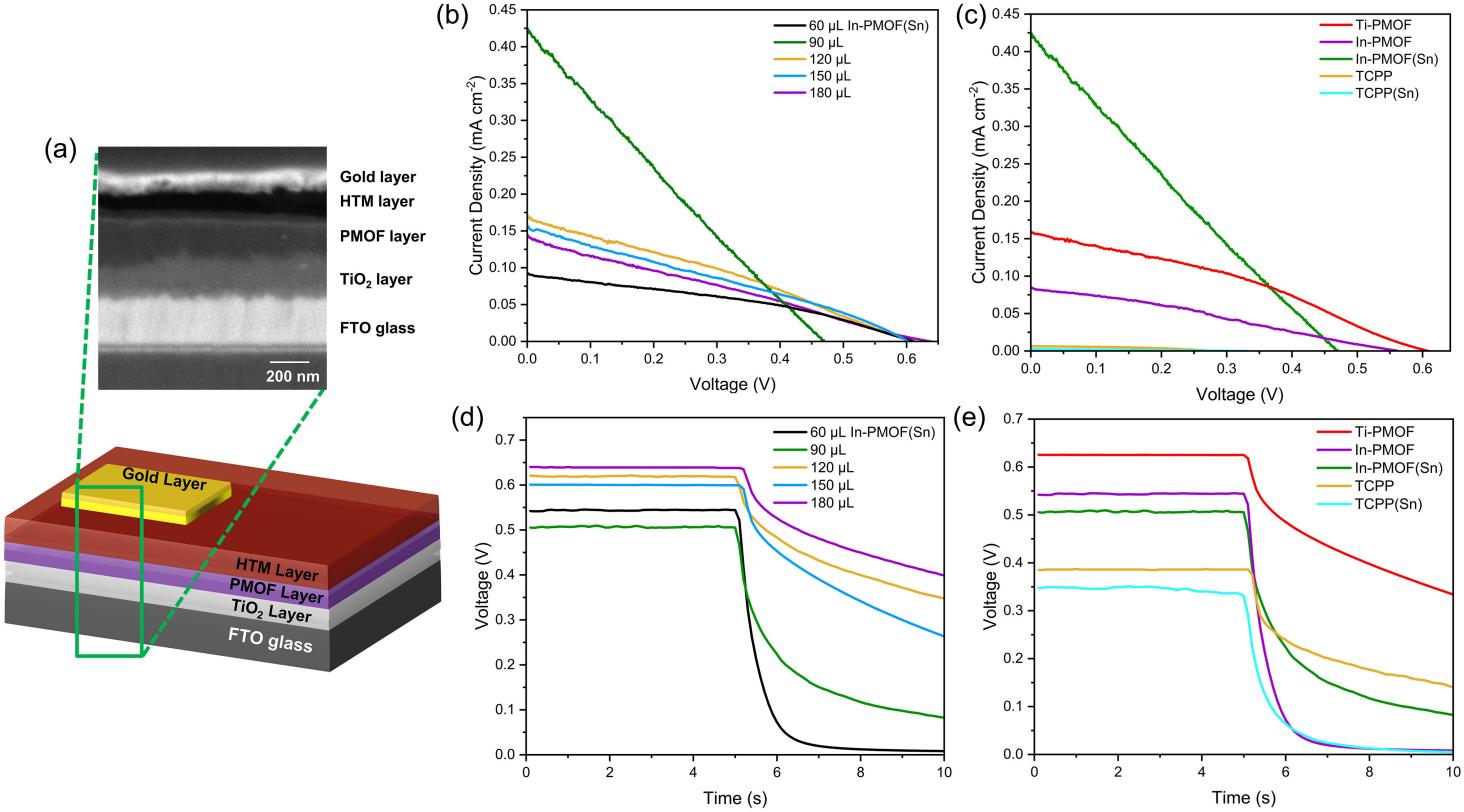
(a) Cross‐sectional SEM image of a representative ssDSSC showing the FTO substrate, TiO_2_ layers, PMOF sensitizer layer, hole‐transporting material (HTM), and gold counter electrode. (b) Representative current density–voltage (*J*–*V*) curves of ssDSSCs incorporating different loadings of In‐PMOF(Sn) under AM 1.5G illumination (60−180 µL of 0.1 mM MOF suspension). (c) Representative *J*–*V* curves of ssDSSCs sensitized with Ti‐PMOF, In‐PMOF, In‐PMOF(Sn), TCPP, and TCPP(Sn) (90 µL of 0.1 mM PMOF suspensions or porphyrin solution). (d) Representative open‐circuit voltage decay (OCVD) profiles of ssDSSCs incorporating different loadings of In‐PMOF(Sn) (60−180 µL of 0.1 mM MOF suspension). (e) Representative OCVD profiles of ssDSSCs sensitized with Ti‐PMOF, In‐PMOF, In‐PMOF(Sn), TCPP, and TCPP(Sn) (90 µL 0.1 mM MOF suspensions or porphyrin solutions). Average photovoltaic parameters are summarized in Table S1.

Encouraged by the favorable energetic alignment and electrochemical properties of the PMOFs, ssDSSCs were fabricated using compact and mesoporous TiO_2_ layers on FTO substrates, followed by BDC anchoring, PMOF deposition, HTM coating, and gold sputtering (Figures 1b and 2). Cross‐sectional SEM imaging of the completed solar cell confirmed successful incorporation of each functional layer (Figure 5a). Representative *J*–*V* characteristics are shown in Figure 5b,c, while the corresponding average photovoltaic parameters obtained from 3 to 5 independently fabricated devices are summarized in Table S1.

The influence of PMOF loading was first examined using varying volumes (60–180 μL) of 0.1 mM In‐PMOF(Sn) suspension in the cell assembly. The representative *J*–*V* curves (Figure 5b) reveal that an optimal deposition volume of 90 μL (∼9 × 10^−8^ mol PMOF) yielded the highest average PCE of 0.0321 ± 0.0068%, with *V*_oc_ = 0.438 V, *J*_sc_ = 0.369 mA cm^−2^, and FF = 0.325. At lower MOF loading (60 μL), lower PCE (0.0135 ± 0.0016%) was observed, which can be attributed to incomplete surface coverage and insufficient light harvesting. At higher MOF loadings (≥120 μL), efficiency also decreased due to likely framework aggregation, increased film thickness, and enhanced recombination losses. Excess PMOF may obstruct TiO_2_ pores, lengthen electron transport pathways, and increase series resistance. The tradeoff between *V*_oc_ and *J*_sc_ observed at higher PMOF loadings suggests increased recombination, likely resulting from longer charge transport pathways within the thicker sensitizer layer.

Using the optimized deposition amount (∼9 × 10^−8^ mol PMOF), Ti‐PMOF, In‐PMOF, and In‐PMOF(Sn) devices were fabricated, and their photovoltaic properties were compared (Figure 5c and Table S1). Based on the averaged photovoltaic parameters, In‐PMOF(Sn) exhibited the highest average PCE (0.0321 ± 0.0068%), followed by Ti‐PMOF (0.0174  ±  0.0061%) and In‐PMOF (0.0115  ±  0.0012%). The superior performance of In‐PMOF(Sn) is primarily attributed to its higher short‐circuit current density, whereas Ti‐PMOF exhibited the highest average open‐circuit voltage. These results indicate that photovoltaic performance is governed by the balance between charge injection, transport, and recombination rather than by any single photovoltaic parameter.

Furthermore, the differences in photovoltaic performance observed between Ti‐PMOF and the In‐based PMOFs are attributed to differences in framework morphology and the resulting balance between charge‐injection efficiency and recombination kinetics at the TiO_2_/PMOF/HTM interface. The larger Ti‐PMOF flakes modify the effective interfacial geometry relative to the smaller In‐based crystallites. While the reduced interfacial contact area may limit the density of charge‐injection sites, it may also suppress recombination by decreasing interfacial back‐electron transfer. The resulting device performance, therefore, reflects the balance between charge injection and recombination arising from the altered interfacial geometry.

Because In‐PMOF and In‐PMOF(Sn) exhibit comparable particle sizes and morphologies, the improved photovoltaic performance of In‐PMOF(Sn) cannot be explained by morphology differences. Instead, the improvement in *J*_sc_ and overall efficiency of In‐PMOF(Sn) relative to In‐PMOF arises primarily from dopant‐induced modifications in redox behavior and interfacial charge‐transfer kinetics. Although the estimated frontier orbital energies remain largely unchanged, the modified redox behavior observed in the CV of the In‐PMOF(Sn) film suggests that Sn incorporation primarily alters interfacial charge‐transfer and recombination kinetics rather than substantially changing the electronic structure of the porphyrin framework, thereby contributing to the enhanced photocurrent observed in the doped system.

To evaluate the role of framework incorporation in ssDSSCs, control devices were fabricated using free TCPP and TCPP(Sn) ligands. As shown in Figure 5c and Table S1, both free porphyrin sensitizers exhibited substantially lower PCEs than their PMOF counterparts. Relative to free TCPP, the In‐PMOF(Sn) device exhibited an approximately sixfold enhancement in average PCE, while the improvement exceeded two orders of magnitude compared with TCPP(Sn). Interestingly, metallation of the free porphyrin with Sn significantly reduced device performance, whereas incorporation of the same Sn‐metallated porphyrin into the PMOF framework produced the highest‐performing device in this study. The contrasting performances of TCPP(Sn) and In‐PMOF(Sn) further demonstrate that the influence of Sn incorporation depends strongly on the framework environment rather than the dopant alone.

To further contextualize the present results, representative photovoltaic performances of molecular and MOF‐based DSSCs reported in the literature are summarized in Table S2. Although the efficiencies reported here remain below those of state‐of‐the‐art molecular sensitizers, the photovoltaic performance of our PMOF‐sensitized ssDSSCs is comparable to that of the limited number of previously reported PMOF‐based ssDSSCs. These results, therefore, provide meaningful insight into the influence of framework composition and dopant incorporation in an emerging class of framework‐based sensitizers rather than representing an optimization study. The mechanistic origins of the observed performance differences are examined in the following section through OCVD and quantitative electron lifetime analyses.

### 3.4 Charge Recombination and Electron Lifetime Analysis

To probe interfacial recombination dynamics, OCVD measurements were conducted (Figure 5d,e), and electron lifetimes (*τ*_*n*_) were subsequently extracted from the voltage decay profiles (Figure S4). Upon illumination switch‐off, the decay of the open‐circuit voltage reflects the lifetime of photogenerated electrons in the TiO_2_ conduction band, with slower voltage decay corresponding to suppressed back‐electron transfer and interfacial recombination. The resulting *τ*_*n*_ profiles (Figure S4) provide a quantitative comparison of recombination kinetics among the different PMOF‐ and free‐porphyrin‐sensitized ssDSSC devices.

For In‐PMOF(Sn) devices, the OCVD measurements (Figure 5d) and the corresponding extracted electron lifetimes (Figure S4) reveal that recombination dynamics depend strongly on PMOF loading. Thinner films exhibited faster voltage decay, consistent with increased recombination due to incomplete surface PMOF coverage and less effective interfacial contact (Figure 5d). At excess loading (180 μL), the slower decay indicates prolonged electron retention; however, the corresponding reduction in *J*_sc_ suggests that charge transport limitations and reduced light penetration offset the benefits of suppressed recombination. These results indicate that optimal device performance arises from balancing charge injection, charge transport, and recombination rather than maximizing electron lifetime alone.

Among the different PMOF sensitizers, Ti‐PMOF exhibited the slowest voltage decay at optimal loading (Figure 5e). The corresponding electron lifetime analysis (Figure S4) further indicates that Ti‐PMOF maintains relatively long electron lifetimes over much of the measured voltage range. This behavior is consistent with its larger flake‐like morphology, which can reduce the effective interfacial contact between the sensitizer and mesoporous TiO_2_, therefore reducing the effective density of interfacial recombination pathways. Nevertheless, Ti‐PMOF did not exhibit the highest photovoltaic efficiency, indicating that reduced recombination alone is insufficient to maximize device performance.

Furthermore, the OCVD measurements (Figure 5e), together with the corresponding electron lifetime analysis (Figure S4), reveal distinct recombination behavior for In‐PMOF and In‐PMOF(Sn), despite their comparable particle sizes and morphologies. These observations further support the conclusion that the enhanced performance of In‐PMOF(Sn) arises primarily from dopant‐induced changes in redox behavior and interfacial charge‐transfer kinetics rather than morphological differences. Because the estimated HOMO and LUMO energies change only minimally upon Sn incorporation (Figure 4b), the combined electrochemical, electron lifetime, and photovoltaic results further indicate that the performance enhancement is governed predominantly by modified interfacial recombination kinetics rather than substantial changes in energetic alignment.

Comparison with devices incorporating free TCPP and TCPP(Sn) further highlights the role of framework confinement. In particular, TCPP(Sn) exhibits a substantially faster voltage decay than In‐PMOF(Sn), consistent with its poor photovoltaic performance (Figure 5e). Although TCPP exhibits slower voltage decay than TCPP(Sn), incorporation of the porphyrin chromophores into the PMOF framework results in markedly improved photovoltaic performance (Table S1), suggesting that framework organization promotes more favorable interfacial charge transfer while mitigating aggregation‐related recombination losses.

### 3.5 Mechanistic Implications

Although the absolute PCE values remain modest for the PMOF‐based ssDSSCs studied, the observed trends provide insight into how framework composition, morphology, and structural organization govern charge injection, transport, and recombination processes in ssDSSCs. The improved performance of PMOF‐based devices relative to free porphyrins confirms that structural confinement within the crystalline framework enhances interfacial organization, facilitates charge transport, and mitigates aggregation‐induced recombination. In contrast to molecular dyes that can adopt random orientations and form uncontrolled aggregates on TiO_2_, the periodic architecture of PMOFs enforces the spatial organization of porphyrin chromophores, likely improving electronic coupling at the TiO_2_ interface and reducing aggregation‐induced trap formation.

The comparative investigation of Ti‐PMOF, In‐PMOF, and In‐PMOF(Sn) demonstrates that morphology and dopant incorporation influence device performance through different mechanisms. The larger Ti‐PMOF crystallites primarily modify interfacial geometry and the balance between charge injection and recombination, whereas the enhanced performance of In‐PMOF(Sn) originates from dopant‐induced changes in redox behavior and interfacial recombination kinetics despite minimal changes in framework morphology and frontier orbital energies.

Collectively, these results demonstrate that framework confinement, morphology, and dopant engineering provide complementary approaches for controlling interfacial charge‐transfer processes in PMOF‐sensitized ssDSSCs. The systematic comparison presented here illustrates that framework morphology primarily governs interfacial geometry, whereas dopant incorporation modifies recombination kinetics without substantially altering energetic alignment. These findings establish a general design strategy for engineering framework‐based sensitizers through independent structural and electronic tuning.

## 4 Conclusion

Titanium‐ and indium‐based porphyrin MOFs (PMOFs) were systematically evaluated as sensitizers in ssDSSCs to distinguish the structural and electronic factors governing device performance. Although all PMOFs exhibited favorable energetic alignment with TiO_2_ and the hole‐transporting materials (HTM), distinct mechanisms were found to govern their photovoltaic behavior. The larger crystallite morphology of Ti‐PMOF reduced interfacial recombination, as reflected by extended electron lifetimes, demonstrating that morphology influences device performance through the balance between charge injection and recombination. In contrast, the enhanced performance of In‐PMOF(Sn) relative to In‐PMOF is attributed primarily to dopant‐induced modifications in redox behavior and interfacial charge‐transfer kinetics rather than substantial changes in frontier orbital energies. The contrasting behavior of the free TCPP(Sn) ligand and the In‐PMOF(Sn) framework further demonstrates that the beneficial effects of Sn incorporation depend strongly on the framework environment.

These findings demonstrate that framework morphology and dopant engineering provide complementary strategies for tuning charge injection, transport, and recombination in PMOF‐sensitized ssDSSCs. By decoupling morphological influences from dopant‐driven kinetic effects, this work establishes clear structure–kinetics–performance relationships that provide a foundation for the rational design of framework‐based sensitizers. Continued advances in controlled film deposition, compositional design, and interfacial engineering may further improve charge‐transfer efficiency and expand the potential of PMOFs for solid‐state photovoltaic applications.

## Funding

This study was supported by National Science Foundation (Grant HRD‐2112554 and DMR 2425229), U.S. Department of Energy (Grant DE‐SC0024498 and DEAC02‐07CH11358).

## Conflicts of Interest

The authors declare no conflicts of interest.

## Supporting information

Supporting Information includes instrumentation, MOF precursor synthesis, solution preparation for solar cell assembly, cyclic voltammetry measurements of PMOF films, determination of HOMO and LUMO energy levels, as well as supplementary figures and tables. The authors have cited additional references within the Supporting Information [23, 30, 32, 39, 45, 52].

## Data Availability

The data that supports the findings of this study are available in the supplementary material of this article.
