# Supplementary material for: Electronic and Structural Effects in Porphyrin Metal–Organic Framework Sensitizers for Solid‐State Dye‐Sensitized Solar Cells
Source: Chemphyschem. 2026 Jul 29;27(14):e70508. doi: 10.1002/cphc.70508 (PMC13421032; doi:10.1002/cphc.70508)
Supplement: Supplementary file 1 — Supporting Information includes instrumentation, MOF precursor synthesis, solution preparation for solar cell assembly, cyclic voltammetry measurements of PMOF films, determination of HOMO and LUMO energy levels, as well as supplementary figures and tables. The authors have cited additional references within the Supporting Information [23, 30, 32, 39, 45, 52]. [file CPHC-27-e70508-s001.pdf]

Electronic Supplementary Information

Electronic and Structural Effects in Porphyrin Metal–Organic Framework Sensitizers for Solid-State Dye-Sensitized Solar Cells

Tommy Taing,[a] Jesus Corona,[a] Sailaja Muduganti,[b] Dr. Xiyuan Yao,[a] Matthew Tang,[a] Doroteo Manriquez,[a] Crystal Xiong,[b] Dr. Oscar O. Bernal,[b] Dr. Lun An,[c] Dr. Long Qi,[c*] Dr. Dianlu Jiang,[a*] and Dr. Yangyang Liu[a*]

[a] Department of Chemistry and Biochemistry, California State University, Los Angeles, 5151 State University Drive, Los Angeles, California 90032, United States

[b] Department of Physics and Astronomy, California State University, Los Angeles, 5151 State University Drive, Los Angeles, California 90032, United States

[c] U.S. DOE Ames National Laboratory, Iowa State University, 2408 Pammel Dr., Ames, Iowa 50011, United States

* *Email:* *yliu114@calstatela.edu*; *djiang4@calstatela.edu*; *lqi@iastate.edu*.

Table of Contents

Section S1. Instrumentation... S2
Section S2. MOF Precursor SynthesisS2
Section S3. Solution Preparation for the Solar Cell Assembly S3
Section S4. Cyclic Voltammetry Measurements of PMOF Films S3
Section S5. Determination of HOMO and LUMO Energy Levels S4
Section S6. Supplementary Figures and Tables S5

Section S1. Instrumentation

Nitrogen adsorption–desorption isotherms at 77 K and Brunauer–Emmett–Teller (BET) surface areas were measured using a Micromeritics ASAP 2020 Plus system. UV–Vis spectra were recorded on a Shimadzu UV-2600 spectrophotometer equipped with an ISR-2600 integrating sphere using 1 cm quartz cuvettes, with a scan interval of 1 nm. Powder X-ray diffraction (PXRD) patterns were collected on a Bruker D2 Phaser diffractometer equipped with a Cu sealed tube (λ = 1.54178 Å), operated at 30 kV and 10 mA. Data were acquired over a 2θ range of 2–20° with a step size of 0.02° and a counting time of 5 s per step. Scanning electron microscopy (SEM) images were obtained using an FEI Quanta FEG 250 field-emission scanning electron microscope. TiO_2 and hole-transporting material (HTM) layers were deposited using a Metrohm Autolab motor controller and spin coater at 2000 rpm. Etched FTO substrates were plasma-cleaned using a Harrick Plasma PDC-32G plasma cleaner under high-power conditions. Gold counter electrodes were deposited using a Cressington 108 auto sputter coater under argon at 5 psi for one sputtering cycle. Electrochemical measurements were performed using a CHI 610 electrochemical analyzer. Cyclic voltammetry was conducted over a potential range of 0–1.6 V (positive sweep) at a scan rate of 100 mV s^{-1}. Current–voltage (J–V) measurements were obtained by linear sweep voltammetry under simulated AM 1.5G illumination provided by a 66001 xenon arc lamp (ORIEL) with a 68811 power supply, calibrated to 0.137 W cm^{-2} with an active area of 0.085 cm^2.

Section S2. MOF Precursor Synthesis

Synthesis of Titanium Clusters for Ti-PMOF. The titanium cluster $Ti_6O_6(O^iPr)_6(abz)_6$ was synthesized according to a previously reported procedure.[1,2] 4-aminobenzoic acid (192.1 mg) and titanium(IV) isopropoxide (103.6 μL) were dissolved in isopropanol (6 mL). The reaction mixture was stirred for 30 min, sealed in a glass tube, and heated at 100 ºC for 77 h. The resulting yellow crystalline product was washed with isopropyl alcohol and acetone, then dried under vacuum at 60 ºC overnight.

Synthesis of TCPP(Sn). The metalloporphyrin TCPP(Sn) was synthesized following a previously reported method (see **Scheme S1**). The reaction afforded a dark purple solid as the final product.[3]

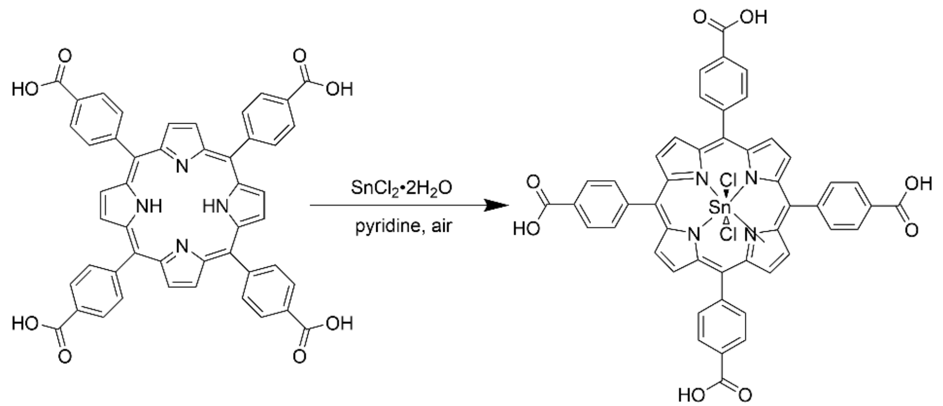

Scheme S1. Synthesis of TCPP(Sn) via tin(IV) metalation of TCPP using $SnCl_2 \cdot 2H_2O$ in pyridine.

Section S3. Solution Preparation for Solar Cell Assembly

TiO_2 Compact Layer Solution. TiO_2 compact layer solution was prepared by mixing isopropanol (6 mL), titanium(IV) *n*-butoxide (0.9 mL), and ethanolamine (0.19 mL) in a sealed microwave vial. The mixture was stirred at 40°C for 2 h until a homogeneous solution was obtained. The solution was stored at 4°C prior to use.

TiO_2 Colloid Layer Solution. The nanocrystalline TiO_2 colloid solution was prepared by combining titanium(IV) *n*-butoxide (12.5 mL) with isopropanol (4 mL). 0.1 M HNO_3 solution (150 mL) was added dropwise to the solution under continuous stirring. The resulting slurry was then stirred at 80°C for 8 h and then transferred to a Teflon-lined solvothermal autoclave at 200°C for 12 h to obtain the TiO_2 colloid.

Anchor Solution. The 0.01 M benzene-1,4-dicarboxylic acid (BDC) anchor solution was prepared by dissolving BDC (166 mg) and KOH (112 mg) in anhydrous ethanol (100 mL) via continuous stirring at 70 °C for 4 h.

Hole Transporting Material (HTM) Solution. The HTM solution was prepared inside a glovebox by dissolving Spiro-MeOTAD (72.3 mg), lithium bis(trifluoromethanesulfonyl)imide) (10 mg), Co(III) TFSI salt (10 mg), and *tert*-butyl pyridine (27 µL) in chlorobenzene (1 mL) in a glass vial. The vial was wrapped in aluminum foil, sealed, and sonicated for 20 min. The solution was stored in the glovebox prior to use.

Section S4. Cyclic Voltammetry Measurements of PMOF Films

A 0.1 M solution of tetra-*n*-butylammonium hexafluorophosphate ($TBAPF_6$) in acetonitrile was prepared as the supporting electrolyte for electrochemical measurements of PMOF films. A separate electrolyte solution containing ferrocene in 0.1 M $TBAPF_6$/acetonitrile was used to determine the ferrocene redox potential for correction to the standard hydrogen electrode (SHE) scale.

Thin films of PMOFs were deposited onto the conductive side of FTO glass substrate. A 0.25 cm strip of polyimide tape was applied across the bottom edge to preserve an exposed region for electrode contact. PMOF suspensions (0.1 mM) were drop-cast in 50 µL increments, with 5 min air-drying intervals between sequential depositions. Total deposition volumes ranging from 100 – 1000 µL film were evaluated to optimize film thickness and electrochemical signal clarity. Films prepared with a total deposition volume of 300 µL yielded the most well-defined redox responses and were used for subsequent measurements.

After drying, the polyimide tape was removed, and electrical contact to the working electrode was established using alligator clips. Cyclic measurements were performed in a three-electrode configuration consisting of the PMOF-coated FTO working electrode, a platinum counter electrode, and a pseudo-Ag reference electrode, mounted in a custom holder.

Section S5. Determination of HOMO and LUMO Energy Levels

Solutions of TCPP and TCPP(Sn) (0.1 mM) were prepared in methanol (0.5 mL), and UV-vis absorption spectra were recorded over the range of 300-800 nm. For PMOF measurements, In-PMOF, In-PMOF(Sn), and Ti-PMOF were prepared by dispersing 3 mg of the respective PMOF in 3 mL of methanol. UV-vis spectra were collected over the same wavelength range (300-800 nm) using an integrating sphere to account for scattering from the suspended framework particles.

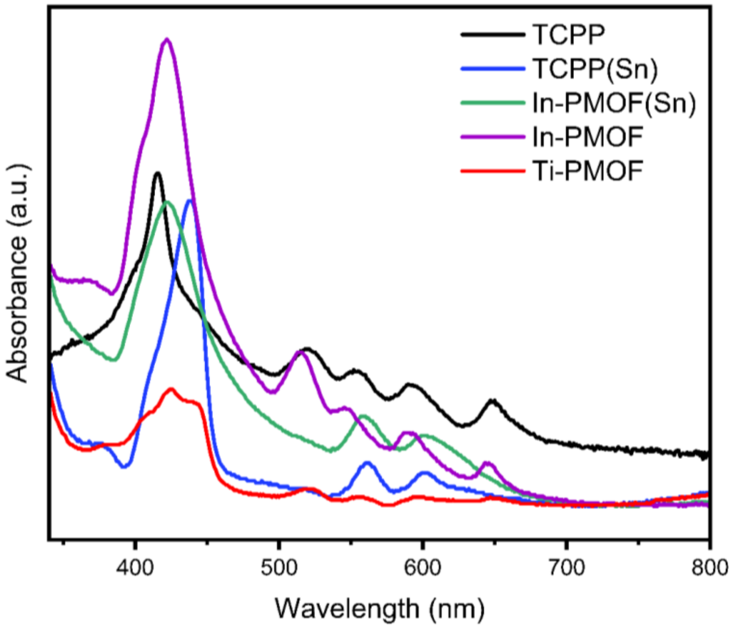

Figure S1. UV-Vis absorption spectra of TCPP (black), TCPP(Sn) (blue), In-PMOF(Sn) (green), In-PMOF (purple), and Ti-PMOF (red).

HOMO and LUMO energy levels were estimated using electrochemical data from cyclic voltammetry (CV) in combination with optical band gap values derived from UV-Vis absorption spectra (**Figure S1**). Electrochemical potentials were referenced to ferrocene and corrected to the standard hydrogen electrode (SHE) scale using $E_{1/2}(Fc/Fc^{+})$ = 0.33 V vs. SHE.

For In-PMOF, the HOMO energy was determined from the half-wave oxidation potential ($E_{1/2}$) of the redox couple: $E_{HOMO} = -\left(E_{1/2,\ ox\ vs\ SHE} + 4.44\right)eV$.[4] The optical band gap (E_g) was determined from the absorption onset using: $E_g = \frac{1240}{\lambda_{onset}}(eV)$. The LUMO energy level was then calculated from: $E_{LUMO} = E_{HOMO} + E_g$.

For Ti-PMOF and In-PMOF(Sn), the oxidation onset potential was used to estimate the HOMO energy: $E_{HOMO} = -(E_{ox\ onset} + 4.44)eV$. The optical band gap and LUMO energy calculations followed the same equations as the In-PMOF.

Section S6. Supplementary Figures and Tables

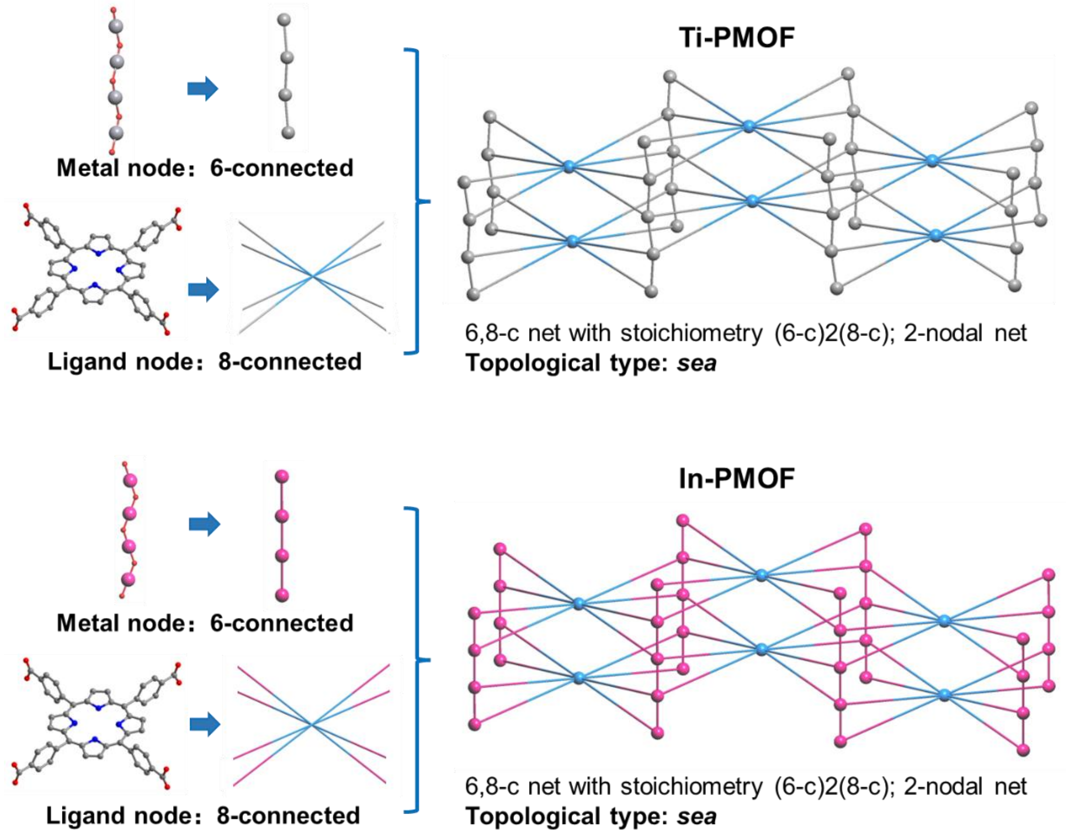

Figure S2. The topological analyses for Ti-PMOF and In-PMOF.

Table S1. Photovoltaic properties of ssDSSCs incorporating different PMOF layers.[i]

MOF	Volume (μL)[ii]	V_{oc} (V)	J_{sc} (mA cm^{-2})	FF	PCE (%)
In-PMOF(Sn)	60	0.455 ± 0.150	0.141 ± 0.047	0.317 ± 0.036	0.0135 ± 0.0016
In-PMOF(Sn)	90	0.438 ± 0.097	0.369 ± 0.15	0.325 ± 0.019	0.0321 ± 0.0068
In-PMOF(Sn)	120	0.519 ± 0.087	0.181 ± 0.023	0.281 ± 0.0073	0.0193 ± 0.0039
In-PMOF(Sn)	150	0.536 ± 0.086	0.157 ± 0.018	0.259 ± 0.021	0.0152 ± 0.0037
In-PMOF(Sn)	180	0.579 ± 0.051	0.123 ± 0.017	0.254 ± 0.013	0.0130 ± 0.0034
In-PMOF	90	0.499 ± 0.063	0.0113 ± 0.033	0.287 ± 0.026	0.0115 ± 0.0012
Ti-PMOF	90	0.508 ± 0.110	0.149 ± 0.024	0.253 ± 0.071	0.0174 ± 0.0061
TCPP	90	0.343 ± 0.073	0.0806 ± 0.018	0.301 ± 0.110	0.00553 ± 0.00097
TCPP(Sn)	90	0.446 ± 0.099	0.00219 ± 0.00048	0.232 ± 0.019	0.000162 ± 0.000038

[i] Values represent the mean ± standard deviation obtained from 3–5 independently fabricated devices (n = 3–5).
[ii] 0.1 mM MOF suspensions were used for deposition; concentration calculated based on porphyrin centers.

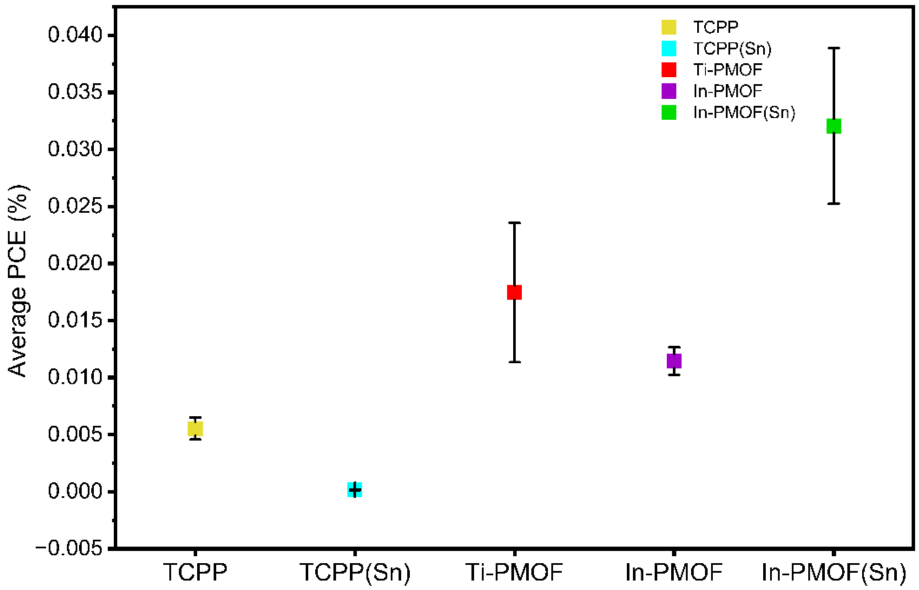

Figure S3. Average power conversion efficiencies (PCEs) of ssDSSCs incorporating TCPP, TCPP(Sn), Ti-PMOF, In-PMOF, and In-PMOF(Sn). Error bars represent one standard deviation obtained from 3–5 independently fabricated devices.

Table S2. Representative molecular and MOF-based sensitizers used in dye-sensitized solar cells (DSSCs) reported in the literature.[i]

Sensitizer	Device Type	Sensitizer Type	J_{sc} (mA cm^{-2})	V_{oc} (V)	FF	PCE (%)	Ref.
**N719**	Liquid DSSC	Ru molecular dye	22.23	1.106	0.847	20.8	[5]
**SM315**	Liquid DSSC	Organic dye	—	—	—	13–29	[6]
**Y123**	ssDSSC	Organic dye	9.8	0.934	0.750	6.9	[7]
**JK2**	ssDSSC	Organic dye	8.9	0.914	0.600	4.9	[7]
**SL10**	Liquid DSSC	Porphyrin dye	17.8	1.040	0.821	15.2	[8]
**Ru-MOF**	Liquid DSSC	MOF sensitizer	2.56	0.63	0.63	1.22	[9]
**Co-DAPV**	ssDSSC	MOF sensitizer	4.92	0.67	0.57	2.10	[10]
**Zn(II)-SURMOF-2**	ssDSSC	PMOF sensitizer	0.053	0.86	—	0.017	[11]
**PCN-222**	Liquid DSSC	PMOF sensitizer	10.97	0.551	0.729	4.41	[12]
**PPF MSSC**	Liquid DSSC	PMOF sensitizer	0.085	0.515	0.521	0.0023	[13]
**Ti-PMOF**	ssDSSC	PMOF sensitizer	0.149 ± 0.024	0.508 ± 0.110	0.253 ± 0.071	0.0174 ± 0.0061	This work
**In-PMOF**	ssDSSC	PMOF sensitizer	0.113 ± 0.033	0.499 ± 0.063	0.287 ± 0.026	0.0115 ± 0.0012	This work
**In-PMOF(Sn)**	ssDSSC	PMOF sensitizer	0.369 ± 0.150	0.438 ± 0.097	0.325 ± 0.019	0.0321 ± 0.0068	This work

[i] Values reported in this work represent the mean ± standard deviation obtained from 3−5 independently fabricated devices (n = 3−5). Literature values were reproduced from the cited references and correspond to the photovoltaic performances reported under their respective experimental conditions.

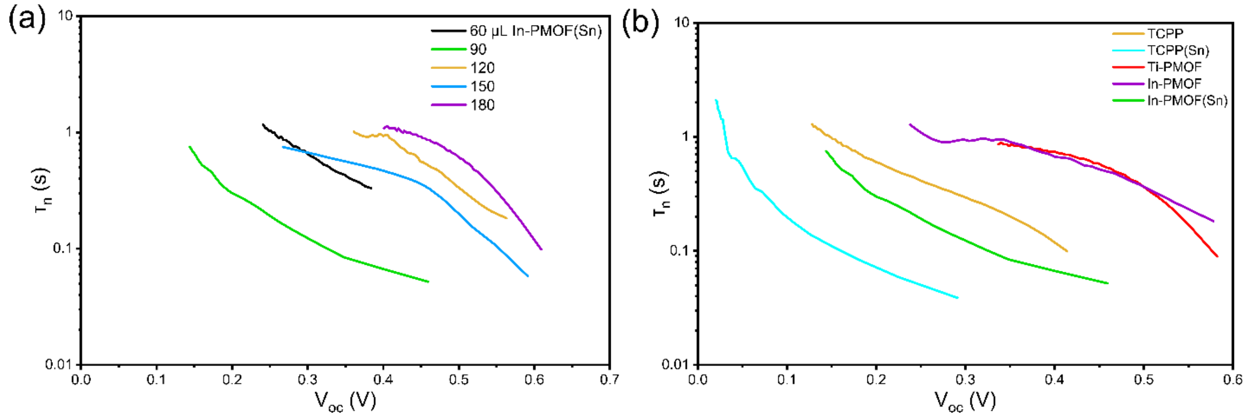

Figure S4. (a) Electron lifetimes (τ_n) extracted from representative OCVD measurements of ssDSSCs sensitized with (a) Ti-PMOF, In-PMOF, In-PMOF(Sn), TCPP, and TCPP(Sn) (90 μL of 0.1 mM PMOF suspension or equivalent porphyrin concentration) and (b) different loadings of In-PMOF(Sn) (60–180 μL of 0.1 mM PMOF suspension)

References

[1] K. Hong, H. Chun, “Nonporous Titanium–Oxo Molecular Clusters That Reversibly and Selectively Adsorb Carbon Dioxide” *Inorg. Chem.* **2013**, *52*, 9705–9707.

[2] Y. Keum, S. Park, Y. Chen, J. Park, “Titanium-Carboxylate Metal-Organic Framework Based on an Unprecedented Ti-Oxo Chain Cluster” *Angew. Chem. Int. Ed.* **2018**, *57*, 14852–14856.

[3] A.-M. Manke, K. Geisel, A. Fetzer, P. Kurz, “A water-soluble tin(IV) porphyrin as a bioinspired photosensitiser for light-driven proton-reduction” *Phys Chem Chem Phys* **2014**, *16*, 12029–12042.

[4] S. Trasatti, “The absolute electrode potential: an explanatory note (Recommendations 1986)” *Pure Appl. Chem.* **1986**, *58*, 955–966.

[5] G. G. Njema, A. Elmelouky, E. L. Meyer, N. Riouchi, J. K. Kibet, “Pioneering an Innovative Eco-Friendly N719 Dye-Sensitized Solar Cell through Modelling and Impedance Spectroscopy Analysis for Energy Sustainability” *Glob. Chall.* **2025**, *9*, e00276.

[6] H. Kaur, N. Goel, “Beyond SM315: A Comprehensive Analysis of Power Conversion Efficiency in Tailored Donor–Acceptor Porphyrin Sensitizers for DSSCs” *J. Phys. Chem. C* **2024**, *128*, 13410–13421.

[7] A. Dualeh, F. De Angelis, S. Fantacci, T. Moehl, C. Yi, F. Kessler, E. Baranoff, M. K. Nazeeruddin, M. Grätzel, “Influence of Donor Groups of Organic D−π–A Dyes on Open-Circuit Voltage in Solid-State Dye-Sensitized Solar Cells” *J. Phys. Chem. C* **2012**, *116*, 1572–1578.

[8] Y. Ren, D. Zhang, J. Suo, Y. Cao, F. T. Eickemeyer, N. Vlachopoulos, S. M. Zakeeruddin, A. Hagfeldt, M. Grätzel, “Hydroxamic acid pre-adsorption raises the efficiency of cosensitized solar cells” *Nature* **2023**, *613*, 60–65.

[9] D. Y. Lee, E.-K. Kim, C. Y. Shin, D. V. Shinde, W. Lee, N. K. Shrestha, J. K. Lee, S.-H. Han, “Layer-by-layer deposition and photovoltaic property of Ru-based metal–organic frameworks” *RSC Adv.* **2014**, *4*, 12037.

[10] D. Y. Ahn, D. Y. Lee, C. Y. Shin, H. T. Bui, N. K. Shrestha, L. Giebeler, Y.-Y. Noh, S.-H. Han, “Novel Solid-State Solar Cell Based on Hole-Conducting MOF-Sensitizer Demonstrating Power Conversion Efficiency of 2.1%” *ACS Appl. Mater. Interfaces* **2017**, *9*, 12930–12935.

[11] J. Liu, W. Zhou, J. Liu, Y. Fujimori, T. Higashino, H. Imahori, X. Jiang, J. Zhao, T. Sakurai, Y. Hattori, W. Matsuda, S. Seki, S. K. Garlapati, S. Dasgupta, E. Redel, L. Sun, C. Wöll, “A new class

of epitaxial porphyrin metal–organic framework thin films with extremely high photocarrier generation efficiency: promising materials for all-solid-state solar cells" *J. Mater. Chem. A* **2016**, *4*, 12739–12747.
[12] J. Jin, "Low-temperature fabrication of metal-organic-frameworks/TiO_2 photoanode for flexible dye-sensitized solar cells" *Mater. Sci. Eng. B* **2024**, *310*, 117755.
[13] E. D. Spoerke, L. J. Small, M. E. Foster, J. Wheeler, A. M. Ullman, V. Stavila, M. Rodriguez, M. D. Allendorf, "MOF-Sensitized Solar Cells Enabled by a Pillared Porphyrin Framework" *J. Phys. Chem. C* **2017**, *121*, 4816–4824.
